# Partial Left Ventriculectomy: Have Well-Succeeded Cases and Innovations in the Procedure Been Observed in the Last 12 Years?

**DOI:** 10.5935/1678-9741.20150061

**Published:** 2015

**Authors:** José Sérgio Domingues, Marcos de Paula Vale, Marcos Pinotti Barbosa

**Affiliations:** 1 Department of Mechanical Engineering of the Universidade Federal de Minas Gerais (UFMG), Belo Horizonte, MG, Brazil and Department of Mathematics of the Instituto Federal de Minas Gerais (IFMG), Formiga, MG, Brazil.; 2 Hospital Nossa Senhora das Dores, Itabira, MG, Brazil.; 3 Department of Mechanical Engineering of the Universidade Federal de Minas Gerais (UFMG), Belo Horizonte, MG, Brazil.

**Keywords:** Cardiomyopathy, Dilated, Cardiovascular Diseases, Hypertrophy, Left Ventricular

## Abstract

**OBJECTIVE:**

In 1996, the Brazilian cardiovascular surgeon, Dr. Randas Batista, introduced a surgical technique called partial left ventriculectomy, where he admitted the possibility of reducing the diameter of the left ventricle through the sectioning of one section of its wall. After the publication of this study, thousands of case reports and procedure analysis have been published, and due to several disappointing results, many doctors and institutions failed to execute it. As the main objective of this study, stands out the search for success cases of ventriculectomy in the last 12 years and if during this period it was achieved some significant development in this procedure that allows obtaining lower mortality rate postoperatively.

**METHODS:**

Systematic review of indexed scientific literature over the past 12 years and the term "Partial Left Ventriculectomy".

**RESULTS:**

There has been a considerable number of reported successful cases and highly significant findings in regard to determining the most suitable region for the section, proper selection of the patients indicated to the procedure, including the influence of the coronary artery anatomy in the nomination procedure and the need for preservation of ventricular geometry to ensure better quality of ventricular contractions after the sectioning.

**CONCLUSION:**

This surgical procedure has been successfully performed, mainly in Japan, improvements in its efficiency were found and the need for a mathematical modeling of the slice to be severed is a prominent factor in many studies.

**Table:** 

**Abbreviations, acronyms & symbols**	
DCM	= Dilated cardiomyopathy
EF	= Ejection fraction
FS	= Fractional shortening
LV	= Left ventricle
NYHA	= New York Heart Association
PLV	= Partial left ventriculectomy
RMV	= Repair of the mitral valve

## INTRODUCTION

Heart problems are responsible for about a third of deaths in the world, so the more we study and understand the structure and functioning of the heart, the greater the chances of obtaining and analyzing new techniques for treatment and prevention of heart disease.

Among these diseases, there is the Dilated Cardiomyopathy (DCM), which is a heart muscle disease characterized mainly by left ventricular (LV) dilatation and systolic dysfunction^[1,2]^. Thus, the ventricles are unable to pump enough blood volume to meet the metabolic needs of the body, leading to heart failure (HF).

Batista et al.^[3]^ present the development of a technique called Partial Left Ventriculectomy (PLV), also known as Batista's surgery, changing the way they considered cardiac interventions when admitted the possibility of reducing the diameter of the left ventricle through the section of a slice of its wall. The procedure is based on the knowledge that in DCM occurs the increasing in LV diameter, but it does not occur sufficient increase in muscle mass, and therefore, a possible damage repair would be the reduction in ventricle diameter to a value close to what was prior to the injury. The operation is based physical and mathematically by one of the forms of Laplace's Law (EQ. 1).

P=kT/R(1)

Where *P* is the intracavity pressure, *T* is the tension on the ventricular wall, *R* is the ventricle radius, *k* is a constant, consisting originally in the section of a muscle slice of the LV lateral wall (generally ellipse shape), starting at the end of it, extending between the papillary muscles and ending close to the mitral ring. The idea is that with the removal of the cardiac muscle slice, a reduction in ventricular diameter can occur, causing the heart to return to being an efficient pump with characteristics similar to that it had before the worsening of the disease.

Once it is a very invasive procedure, the technique is designed to patients in advanced stages of DCM. After Batista's study, thousands of case reports and analyzes of the procedure were published, however, due to several disappointing results, many doctors and institutions ceased to execute it. These bad results show mortality rates ranging from 25% to 80% and a relation with many of these works can be obtained in Lunkenheimer et al.^[4]^.

As the main objective of this study stands out the search for success stories of ventriculectomy in the last 12 years and if during this period it was achieved some significant development in this procedure that allows obtaining lower mortality rate postoperatively.

## METHODS

This is a study of systematic review of literature, developed with indexed scientific production and it has been performed through search in the periodical portal of CAPES.

The search has been made in order to obtain articles with the descriptor "Partial Left Ventriculectomy" anywhere in the text, which have been published in any language and in the last 12 years. In addition, the option to display only peer-reviewed articles was selected.

After gathering and obtaining all the studies presented in the survey, an analysis based on abstracts was performed, as well as discussions and conclusions of all of them, in order to consider only items that could help in achieving the real goals of this study. In other words, papers involving reports of PLV success and those involving specific studies or reports to identify ways to improve the effectiveness of this surgical procedure.

## RESULTS AND DISCUSSION

The performed search had collected 206 articles distributed in 22 professional periodicals, allocated in databases: Scopus, MEDLINE, ScienceDirect, OneFile, Web of Science, Scielo and PubMed databases. Moreover, some of them were duplicated, once they appear in more than one database and many had no relation to the stated objectives for this study. Thus, after analysis and rigorous selection, 43 articles were obtained with the desired profiles. The nationalities of the institutions of the authors are reported in Table 1.

**Table 1:** Number (N) of articles and their respective percentages (%), according to the countries of the main authors involved in the research/articles.

Country of the 1^st^ author and his institution	N	%
Japan	20	46.5
Germany	9	20.9
United Kingdom	4	9.3
Brazil	2	4.7
Serbia and Montenegro	2	4.7
Denmark	1	2.3
Italy	1	2.3
Korea	1	2.3
China	1	2.3
Netherlands	1	2.3
Switzerland	1	2.3
Total	43	100

It can be seen the clear predominance of Japan as a leader in publications of work relating to success case studies and developments in the PLV, with 46.5% of the 43 analyzed studies, followed by Germany with 20.9%. From what it can be seen, the Japanese dominance in these studies may have intimate relation with the legal difficulty, because in Japan the organ donation is forbidden for children under 15 years^[5]^. In addition, religious issues also influence the decision making for transplants^[6]^.

The individual participation of Brazil is small, but if added the number of studies published with co-participation of researchers and/or national institutions, there is a total of 8 articles, which represents 18.6% of the analyzed studies, indicating a good Brazilian contribution in this area, including many works with the participation of Dr. Randas himself.

### Case reports

Some success cases have been described over the past 12 years. One of them was the case report of three Japanese children aged between 8 months and 3 years and a half, all with DCM. They had undergone PLV and repair of the mitral valve (RMV) at the Yamanashi Medical University hospital from May 1998 to April 2000^[5]^. The procedure that was performed in the first child was urgently done and after 3 months her condition got worse and she had to be transferred to the US for transplantation, since in Japan organ donation for children under the age of 15 years is not allowed.

The other two children responded well to the procedure and factors such as fractional shortening (FS) and ejection fraction (EF) improved significantly, allowing great improvement in life quality. During a couple of years of postoperative follow-up, all patients presented good recovery.

The authors point out that the area to be sectioned in adults may vary according to the most damaged myocardial region, found by preoperative evaluation. However, this region is narrower in children, once the mitral valve replacement and the papillary muscles translocation are not recommended, thus restricting the section of the heart section to the inter-papillary region. Therefore, this observation may define the best way to select a patient who is a PLV candidate, by choosing the most damaged myocardium region. In addition, only the child who was urgently submitted to procedure has not achieved good results after the surgery, indicating that important factors to the surgery success may not have been analyzed, once the cardiovascular surgery has as its foundation operate, whenever possible, on an elective basis, in order to the larger set of data on the patient can be analyzed and the best decision taken, thereby maximizing the probability of success in the proceedings.

Another interesting study that indicates positive results after completion of the PLV, as the one presented by Sugiyama et al.^[5]^ is the study performed by Coelho de Souza et al.^[7]^. In his study, only one child diagnosed with idiopathic DCM has been undergoing the procedure and monitored for 70 months. This child was the same age range reported in^[5]^ 2 years and a half, and in functional class III/V as the cases analyzed by Batista et al.^[3,8,9]^. After the procedure both EF and the patient's FS of the LV improved significantly, reaching values of 63% and 28%, respectively, 24 months after the PLV. By the fifth year of follow-up these favorable results regressed a little. However, during this entire period the patient was classified in NYHA class I.

Souza et al.^[7]^, in line with Sugiyama et al.^[5]^ believe the procedure should be considered for treatment, especially because it also works as a bridge to transplantation. It also improves significantly the child's life quality over a long period, including greater tolerance to physical activity. Still in agreement, both studies emphasize the importance to develop means to define protocols for each patient, in order to reduce the risks of intraoperative and postoperative complications.

Yamagishi et al.^[10]^ and Westaby et al.^[11]^ present separately a female child case, one is 3 years old and the other is only 5 months old, both with ischemic DCM caused by anomalous origin of the left coronary artery of pulmonary artery, submitted to PLV. In both cases, and in agreement with the results of Souza et al.^[7]^ study, after performing the procedure, the kids have already presented very significant improvements in LV function, sharp reduction in the cavity volume and great improvement in FS. However, only Westaby et al.^[11]^ performed monitoring, and it is reported that in the last assessment the child was already 10 years old, in NYHA class I, played sports and had normal mental and physical development, showing great results at long term.

More encouraging results have been obtained by Horii et al.^[12]^ and Suma et al.^[13-15]^, which unlike Sugiyama et al.^[5]^, Souza et al.^[7]^ and Yamagishi et al.^[10]^, analyzed the results of PLV completion in much larger samples, 70, 96, 107 and 36 patients, respectively, with global aged between 14 and 76 years, all in functional class III or IV by NYHA, and 83% of these also underwent mitral valve reimplantation. The determination of the region to be sectioned was made based on intraoperative echocardiography, by choosing the area which wall was thinner and akinetic. The results obtained by Horii et al.^[12]^ are very similar to those obtained by Suma et al.^[13-15]^, and all corroborate with Sugiyama et al.^[5]^ when they conclude, as it was expected, that the best results were obtained for the procedures performed on an elective basis, whose hospital mortality rates were between 7% and 13.8%, against high rates to those performed on an emergency basis, ranging from 57% to 60.9%. Moreover, the results obtained by Horri et al.^[12]^ for survival rates during the first year, 75.5%, are relatively better than those obtained by Suma et al.^[13-15]^, whose respective rates were 66.4, 66.9 and 67.5%. Besides that, in all studies, good survival rates for subsequent years were presented and 50.2% obtained by Suma et al.^[14]^, the highest 7-year survival rate in these articles. In all these studies, the patients remained in functional class I and II, with increased EF and LV dimensions reduced throughout the follow-up period that lasted seven years in maximum^[12-15]^

In Kawaguchi et al.^[16]^ study, 65 patients underwent LPV, and among them, 24 also underwent RMV. As a result, 83% of the patients (54 patients) have been discharged from hospital and had significant improvements in ventricular dimensions, contractile function, EF and circumferential shortening. Only 37% of them (24 patients) have been monitored during a period of 170±115 days. During this time, significant improvements have remained in all patients.

Giuffrida et al.^[17]^, Soo et al.^[18]^ and Shin et al.^[19]^ reported very interesting cases of major successes in monitoring only one patient in each one of the articles, after the completion of LPV. In the first study a 60-year-old man with DCM and HF of functional class III have been monitored during 40 months and showed considerable progress in EF, which passed from 15% to 30% and he went up to class II, greatly improving his life quality. On the other hand, Soo et al.^[18]^ present the 8-year monitoring of a 65 year-old man with class HF III, submitted to LPV and RMV. There was an overall improvement in ventricular function which remained throughout the follow-up period, leading the HF to be reclassified to Class I and causing the patient to have an amazing ability to exercises.

In line with the results obtained by Soo et al.^[18]^, Shin et al.^[19]^ present a unique monitoring case of a patient undergoing PLV for 13 years (first PLV held in Korea) and performing 3d echocardiography to evaluate the state of the cardiac muscle, its dimensions, volume and EF. The results placed the HF patient in functional class II and demonstrated that the procedure benefits have been the same during the monitoring.

Besides the use of PLV to reduce the tension on the left ventricle wall in patients with DCM, Bossert et al.^[20]^ have described another application of this procedure. In this study, it is reported the use of this technique for the removal of a cardiac fibroma in the free wall of the LV of a child of only eight months. During a 48-month follow-up it has not been observed any recurrence of the tumor and the proper LV functioning was confirmed.

Schäfers et al.^[21]^ and Wilhelm et al.^[22]^ present the results of a 100% successful experiment in performing the PLV with reimplantation of the mitral valve in a group of 12 patients. After all the procedures, they already had significant signs of increase in EF and decrease in LV dimensions. However, 2 patients died within a year of completing the procedure. However, the other 10 patients maintained the good results of the procedures after a year, and the survival rate during this period was then 83.3%. The PLV indication for these patients was carefully made, where the nominees were chosen based on NYHA functional class (>III), cardiac index (<2.5 liters/min/m^2)^, maximum consumption of O_2_ (<14 ml/kg/min), LV final diastolic diameter (>7) and mitral insufficiency (>2).

The PLV has been also successfully tested in dogs, as it can be seen in the study by Christiansen et al.^[23]^, Christiansen & Gruber^[24]^ and Martins et al.^[25]^. The first two studies reported an experiment where DCM was induced in a group of 6 dogs with the use of adriamycin intracoronary following a protocol pre-defined by the researchers. All the dogs were submitted to PLV. The mortality rate was 25% during a 13-week follow-up, where EF and ventricular dimensions had their values improved.

In these studies, until they reach the protocol that led them to a 25% mortality rate, it was reported that in a previous group of dogs where DCM had been induced, there was a 100% of mortality after the PLV. It occurred because the dogs had grand marginal arteries, increasing the probability of myocardial infarction, due to the need for ligation in case of PLV. Therefore, they got to a very important conclusion, once it was observed that the arteries anatomy is an important factor for the PLV success.

In Martins et al.^[25]^ a group of four dogs underwent PLV and the other, also composed by 4 dogs underwent partial right ventriculectomy (PRV), all the animals were not carriers of DCM and they were all examined and studied during the pre-, intra- and postoperatively periods, during 1, 7, 14, 21, 30 and 60 days after surgery, with 100% survival in this period. Due to the excellent obtained results, both studies indicate the procedure as an evolution form to the DCM treatment searches.

In a study on the LPV results in elderly patients, Shimura et al.^[26]^ indicate that they can also receive benefits with this procedure, because the analyzes present results similar to those obtained for younger people, with 57% of hospital survival of the elderly (over 65 years) against 62% of the younger ones.

In 2005 a study about the morphological effect in 15 patients surviving to PLV was performed a year after its completion, and it was verified that in addition to the significant reduction in the functional classes these patients presented, there were also favorable effects on myocardial structure, especially in the ventricular diameter and in the tissue quality^[27]^.

More recently, two studies were published and they reported success in the PLV performing. At first, Yoda et al.^[28]^ describe the case of a patient with DCM that has been subjected to various procedures, including PLV. The 29-year-old man, who was in functional class IV (NYHA) presented significant improvements in the LV function after the procedures had been performed and it took him not much time to go back to normal life (Class I), and these circumstances were kept for throughout the period of one year of monitoring. In the second report, González López et al.^[29]^ present a case similar to those described by Yamagishi et al.^[10]^ and Westaby et al.^[11]^, where a four-month baby with anomalous origin of the left coronary artery of the pulmonary artery and who developed severe heart failure with EF<20%, was submitted to PLV. After the procedure it was obtained EF=55% and reduced to NYHA class I, and these values remained during the period of one year follow-up.

The vast majority of the successful study described herein have in common an important stage which we believe is crucial to the success of the LPV^[5,10-15,18,20,21,25,28,29]^. This stage consists in assessing the best area to be sectioned in the lateral wall of the LV by intraoperative echocardiography (besides, of course, careful visual assessment of myocardial conditions after the heart exposure). In this evaluation, as well as performed in Horii et al.^[12]^ and Suma et al.^[13-15]^, the area to be sectioned in the free wall of the LV, generally was that thinner and akinetic one, since this region does not contribute to the proper functioning of this cavity. Some of the studies did not perform this stage, or at least did not report it^[7,16,17,19,22,23,26]^. In case some of them have not done this assessment, it may be that the sections have been made in kinetic regions, still leaving akinetic regions in the LV wall. In other words, the active parts may have been sectioned and the inactive parts of the ventricles sidewalls may have been left apart. This stage, though the results have been good in all of them, perhaps could assist in obtaining even better results. In addition, it is also unanimous the need for careful selection of patients to be submitted to PLV, which is also corroborated by other studies^[4,30-35]^. However, it is not observed a standard protocol selection for candidates to this procedure, indicating that developments in this area and discussions on this protocol must be made.

### Case reports and possible procedure innovations

Several articles published by leading researchers worldwide have some good ways to improve the PLV efficiency.

Koyama et al.^[36]^ present an innovative study, demonstrating that the LV apex plays a key role in optimizing its functioning. This information was the basis for their experiment on dogs, where two groups, each one composed by six dogs had induced DCM and underwent PLV, and one of the groups had a section made until the apex, which was preserved in the other group. The results of the group where the apex had been preserved were much better. This result allowed new insight into the way of sectioning the heart section in the LV wall, indicating that the apex preservation could improve the results of the PLV. Subsequent studies stressed the importance of this result and confirm their veracity^[37-40]^.

In addition, Matsui et al.^[38]^ also mention the importance of preserving the original ventricular geometry so that its functions can be fully accomplished. Therefore, it is necessary that the section made on its wall allows its permanency on elliptical shape. The importance of preservation or restoration of the original LV geometry is not highlighted only in studies related to PLV, as seen in the studies by Braile et al.^[41]^, Jatene et al.^[42]^, Braile et al.^[43]^ and Ferreira Filho^[44]^.

Important notes on improvement possibilities in the PLV can be analyzed in Lunkenheimer et al.^[45]^, which among other successful researchers, there is also Dr. Randas Batista as a member. In this study, the authors stress that until the moment of its fulfilling, the size, shape and location of the section to be sectioned was the surgeon's decision to make, and once the procedure had been performed, it was impossible to avoid future problems, once removal would have been already made. It also describes that when making an exaggerated section in the LV, the RV function may also be impaired.

The authors also present an experiment report conducted in pigs with a realization protocol of the PLV, in which they obtained good results. However, this study was published almost simultaneously with the study by Koyama et al.^[36]^ and maybe that is the reason why it does not incorporate the need to preserve the apex.

The LV region location where the section should be done may be performed in most of the cases by the volume reduction test based on echocardiography, as already observed in the previous section, which is already a major breakthrough that allowed the best results to the procedure. The format of the section to be sectioned is generally elliptical, but in some cases it has already been performed hourglass shaped sections^[46]^.

However, in spite of considering the original geometry of the LV is best preserved when the section is elliptical, no works were found on studies reporting the influence of the sections formats in the procedure's efficiency and that assist in the maintenance of the LV geometry.

Other studies also report that one of the facts that contribute to the failure of the PLV realization is the myocardial extended section, in other words, the withdrawal of a larger section of what it should be^[16,47-50]^. Kawaguchi et al.^[48]^ report that the ideal is that the section is the minimum size required, while in Westaby^[49]^ it is emphasized that, until the completion of their work, there were no ways to determine the optimal amount to be sectioned from the LV wall, allowing the removal of exaggerated sections, impairing the ventricular function. On the other hand, Kawaguchi et al.^[16]^ reported that a very long incision may generate ventricular constriction, because the ventricular cavity reduction will be bigger than necessary, making the heart vulnerable to ventricular fibrillation or sudden death.

Aiming to obtain a method of determining the size of the section to be sectioned, and therefore allowing that the problems related to myocardial extended section may be avoided, a research group of the Post-Graduation in Mechanical Engineering/Bioengineering at the Universidade Federal de Minas Gerais (Federal University of Minas Gerais) has started a research on the possibilities of determining these dimensions, and preliminary theoretical results have been published in major conferences in this area^[51-53]^. It is believed that after the consolidation of the way to obtain these dimensions, it should be a mandatory part of the realization protocol of PLV, where each patient will quickly have the determination of how much must be sectioned so that the removed section is actually the ideal size.

Three mathematical models related to PLV have been found. Ottesen & Danielsen^[54]^ present a model to make the analysis of the ventricular contraction, and it can be used to measure the PLV efficiency, comparing prior and subsequent results to the procedure. Dorri et al.^[35]^ developed a model to investigate the ventricular deformation and used it to prove that in properly selected cases this procedure may be feasible, and that the PLV is according to physical-mathematical description expressed in Batista et al.^[3,9]^. The third model is described by Warwick et al.^[55]^ and it is based on a mathematical technique called "finite element", and it aims to provide changes in LV contractile function ulterior the PLV, to assist in a more adequate selection of the patients for this procedure. However, no reports of further improvements and practical applications of these models were found, indicating that so far, it has been made very little evolution of mathematical modeling application as an assistant in improving and analysis of PLV.

Thus, after all the reports described in this study, it is possible to believe that many of discouraging results on the PLV perhaps could have been avoided if the selection criteria had been better studied, if the determination of the most affected regions of the LV wall had been analyzed and if the results such as the need to preserve the apex and the coronary arteries anatomy have been investigated previously.

## CONCLUSION

During the last 12 years, important reports of success and innovation in the PLV achievement have been published. Japan presents the largest number of achievements and innovations in the procedure and Brazil has a considerable contribution as the country of the lead authors of two studies and participation in more than 18% of the total articles that have been found.

It was evident that the quality of the PLV results can be improved and it is needed a rigorous selection criteria of the patients which best suit to procedure.

The necessity of the ventricular apex preservation when executing the section, in order to ensure the quality of ventricular contractions after the PLV is a prominent factor in this study, and it proved to be very important in a considerable number of the searched studies. Thus, this consideration should always be taken into account while performing the PLV. In addition, another important factor is the anatomy of the coronary arteries, since it has been found that patients with large marginal arteries are at high risk of heart attacks after the PLV.

Many studies show the necessity of making appropriate size sections to prevent excessive reduction of the ventricular cavity. For that reason, a mathematical and computational model, capable of performing sizing of the heart section to be sectioned, and thus may prevent the occurrence of complications related to sections extended, is already being studied and developed.

**Table:** 

**Authors' roles & responsibilities**		
JSD	Analysis/interpretation of data; statistical analysis; final approval of the manuscript; study design; implementation of projects/experiments; manuscript writing/critical review of its content	
MPV	Analysis/interpretation of data; final approval of the manuscript; study design; implementation of projects/ experiments; manuscript writing/critical review of its content	
MPB	Analysis/interpretation of data; final approval of the manuscript; study design; implementation of projects/ experiments; manuscript writing/critical review of its content	
